# Analysis of serum vitamin D and calcium levels in elderly patients with stable and unstable intertrochanteric fractures: A multi-center prospective study

**DOI:** 10.1371/journal.pone.0313023

**Published:** 2024-11-22

**Authors:** Kamran Alijanpour, Sina Afzal, Abolhasan Alijanpour, Hasan Barati, Hemmat Gholinia, Mehdi Tavassoli

**Affiliations:** 1 Department of Orthopedic and Trauma Surgery, Babol University of Medical Sciences, Babol, Iran; 2 Department of Orthopedic and Trauma Surgery, Shahid Beheshti University of Medical Sciences, Tehran, Iran; KPC Medical College and Hospital, INDIA

## Abstract

**Background:**

Hip fractures are highly prevalent in the elderly, with intertrochanteric (IT) fractures of the femur constituting about half of the fractures in this region. We aimed to evaluate the levels of serum calcium and vitamin D in patients with stable or unstable IT fractures to study their contribution to the severity of IT fracture.

**Materials and methods:**

Using a prospective cross-sectional design, we enrolled patients with IT fractures admitted to two referral orthopedic centers in 2022. Unstable IT fracture was defined as those with reverse obliquity or a trans-trochanteric pattern of fracture, a large or comminuted posteromedial fragment, and subtrochanteric extension of the fracture. Serum 25 (OH) vitamin D and calcium levels were the main study variables assessed by lab tests and compared among the two study groups: stable and unstable IT fractures.

**Results:**

A total of 286 patients with a mean age of 70.5 ± 7.3 years and a female predominance (60.4%) were included in the final analysis. Among the patients, 139 (48.6%) had stable IT fractures, and 147 (51.4%) had unstable IT fractures. The mean serum level of vitamin D was significantly higher in the stable group (30.3 ± 7.0) compared to the unstable group (26.5 ± 6.1) (P-value = 0.007). Increasing age was associated with a decreasing level of serum vitamin D; however, the association was weak and not statistically significant (P-value = 0.319). The mean serum calcium level was higher among stable cases (8.6 ± 0.7) compared to unstable cases (8.4 ± 0.9); however, the difference was not statistically significant (P-value = 0.540). Vitamin D and calcium levels were almost similar among males and females.

**Conclusion:**

Lower levels of serum vitamin D were significantly associated with unstable IT fractures, and supplementation with this element might prevent severe fractures of this type and other fragility hip fractures.

## Introduction

Hip fractures are one of the most prevalent types of fractures among the elderly, and with increasing life expectancy and aging populations globally, their incidence has shown an upward trend in recent decades [1]. While there are variations in the epidemiology of hip fractures based on geographical locations and population characteristics worldwide, the increasing trends have been concerning in nearly all countries and regions [2]. Intertrochanteric (IT) fractures of the femur constitute approximately half of the hip fractures in the elderly population, who are vulnerable to frailty, osteoporosis, and a higher risk of falls [3, 4]. In addition to the clinical epidemiology and burden, IT fractures also account for almost half of the economic burden associated with hip fractures, posing a significant strain on healthcare systems and patients affected by this injury [5]. These fractures present various challenges, particularly unstable fractures, which are associated with higher post-operative complications such as non-union, leading to increased mortality and morbidity rates [6]. Unstable IT fractures generally refer to those with a reverse obliquity or trans-trochanteric pattern of fracture, a large or comminuted posteromedial fragment, and subtrochanteric extension of the fracture [7].

The management of IT fractures involves various surgical methods, including intramedullary nails and sliding hip screws, each with its advantages and disadvantages [8]. A study from Iran reported high post-operative complications following the treatment of IT fractures in the elderly population, outcomes associated with factors including instability and the quality of the fractured bone undergoing fixation [9]. Investigations into the factors associated with the incidence and outcomes of IT fractures have led researchers and clinicians’ attention to serum elements contributing to bone health, which may determine the severity of fractures and the success of surgical treatment [10]. Among these elements, vitamin D and calcium play pivotal roles in bone health through various pathophysiological pathways, and studies suggest that appropriate supplementation with these elements can prevent hip fractures and facilitate fracture union [10–12].

Previous literature has examined the association between serum levels of vitamin D and calcium and the severity of hip fractures, indicating that lower serum calcium levels and deficient or insufficient serum vitamin D levels are associated with more severe hip fractures [13, 14]. This finding has prompted consideration of vitamin D supplementation alongside calcium to prevent severe osteoporotic hip fractures in the elderly [14]. However, evidence on the severity of IT fractures, particularly stable and unstable types, is limited, with available literature reporting an association between low vitamin D and calcium levels and unstable IT fractures [15–17]. Nevertheless, these findings are based on small sample sizes and retrospective study designs, making definitive conclusions challenging [15, 16]. Therefore, we designed a study to analyze serum levels of vitamin D and calcium among patients with IT fractures at two referral orthopedic surgery centers in Iran, aiming to investigate the association between serum levels of these elements and the stable or unstable types of IT fracture. The findings of this study may contribute to the existing body of evidence on vitamin D and calcium supplementation in the elderly to prevent severe fragility fractures in this vulnerable population.

## Materials and methods

### Study design and population

This study was designed as a prospective cross-sectional investigation on patients with IT fractures admitted to two referral orthopedic centers, namely Shahid Yahya Nezhad Hospital and Shahid Beheshti Hospital, both affiliated with Babol University of Medical Sciences in Babol, Iran, during the period from January 1^st^ to December 31^st^, 2022. Inclusion criteria comprised individuals aged over 55 years with any type of IT fracture, including both stable and unstable cases, admitted to the study centers during the specified timeframe. Exclusion criteria encompassed patients with femoral head or neck fractures, those who did not provide informed consent, individuals with laboratory test failure, and patients diagnosed with neurological, psychiatric, or metabolic diseases due to their potential impact on surgical and treatment outcomes and the bone healing process. Unstable IT fractures were characterized by reverse obliquity or a trans-trochanteric pattern of fracture, a large or comminuted posteromedial fragment, and subtrochanteric extension of the fracture [6, 7]. The required sample size for this study was determined to be 300 cases of IT fractures. Quota sampling was used to select participants, equally distributing them between stable and unstable fracture categories based on the prevalence of this fracture type in Iran [18].

### Data collection and study variables

Data collection for this study involved the enrollment of patients based on the inclusion criteria at the time of admission, with laboratory values assessed in the pre-operative stage. The main study variables were serum vitamin D and calcium levels, assessed by drawing 5 cc of venous blood and separating serum by centrifugation after coagulation of the blood cells. The 25 (OH) Vitamin D level was determined using an ELISA kit produced by Ideal Tashkhis Atieh Company (Iran), with a normal value range defined as 30–70 ng/ml. Serum total calcium was assessed using the Arsenazo III calcium-sensitive dye and photometric method by the Pars Azmoon kit (Iran), with the normal range for its value defined as 8.6–10.3 mg/dl. Patients’ age and sex were demographic variables used to compare study outcomes across different stratifications based on the type of IT fracture (stable/unstable).

### Statistical analysis

Quantitative variables were presented as mean with standard deviation (±SD), while qualitative variables were summarized in terms of frequency and percentages. The associations between different categorical and quantitative variables were assessed using the chi-squared test and independent samples T-test. Pearson correlation coefficient was employed to evaluate the degree of association between two quantitative variables. To ensure the robustness of the study findings, a post-hoc power analysis was conducted based on the mean values of serum levels of vitamin D and calcium as continuous endpoints of the study, with the sample size distributed across two study groups as the two independent samples, and considering a type I error of 0.05. A P-value <0.05 was deemed statistically significant for all tests conducted. Data analysis was performed using IBM SPSS Statistics for Windows, Version 21.0 (Released 2012; Armonk, NY: IBM Corp.).

### Ethical considerations

This study obtained ethical approval from the ethical committees at Babol University of Medical Sciences, Babol, Iran (IR.MUBABOL.REC.1400.211). All patients provided informed written consent before participating in the study and agreed to the publication of results. Study investigators were dedicated to safeguarding the privacy of patients throughout the process of data collection, analysis, and reporting.

## Results

### General findings

A total of 300 patients with IT fractures were initially enrolled for this study. However, 14 patients were excluded due to pre-determined exclusion criteria, resulting in a final sample size of 286 patients included in the analysis. Among these patients, 173 (60.4%) were female, while 113 (39.6%) were male. The mean age of the patients was 70.5 ± 7.3 years. In terms of fracture pattern, 139 (48.6%) were classified as stable IT fractures, while 147 (51.4%) were categorized as unstable IT fractures (Table 1).

**Table 1:** Baseline characteristics of the study participants and the results of laboratory measurements.

Variable	Study group		P-value
	Stable fracture (N = 139)	Unstable fracture (N = 147)	
Sex, No. (%)			0. .984
Male	55 (48.7%)	58 (51.3%)	
Female	84 (48.5%)	89 (51.5%)	
Age, mean ± SD, year	69.7 ± 7.9	71.4 ± 6.5	0.326
Vitamin D, mean ± SD, ng/ml	30.3 ± 7.0	26.5 ± 6.1	**0.007**
Calcium, mean ± SD, mg/dl	8.6 ± 0.7	8.4 ± 0.9	0.540

SD: standard deviation

### Vitamin D and calcium assessments

The mean serum vitamin D level was significantly higher in the stable group (30.3 ± 7.0) compared to the unstable group (26.5 ± 6.1), with the difference reaching statistical significance (P-value = 0.007). Vitamin D levels were similar between males (28.7 ± 6.7) and females (28.2 ± 6.8) (P-value = 0.727). Increasing age was weakly associated with a decrease in serum vitamin D levels; however, this association was not statistically significant (Pearson correlation coefficient: -0.106, P-value = 0.319).

**Fig 1:**
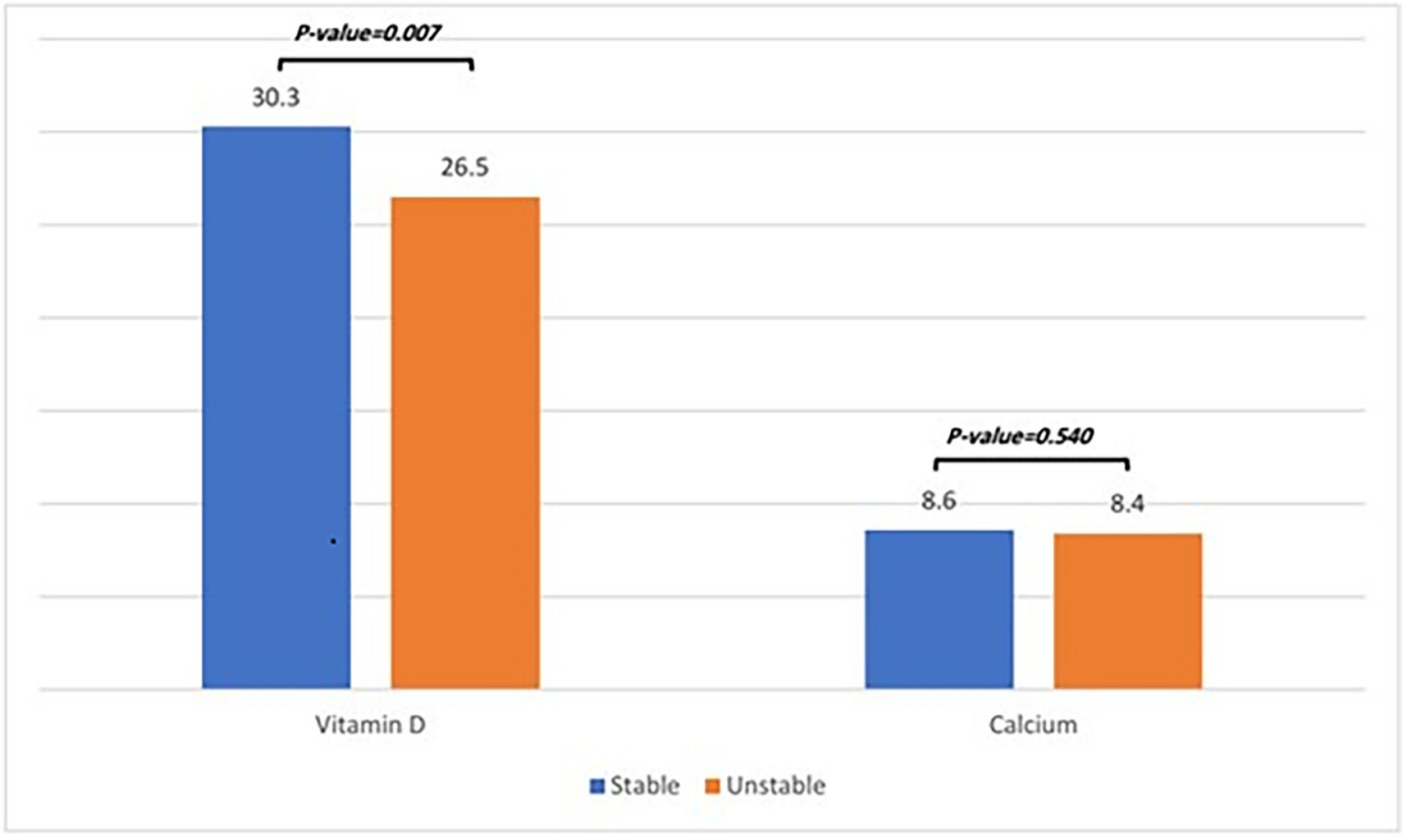
Comparison of serum vitamin D and calcium levels in patients with stable and unstable intertrochanteric fractures included in this study.

Regarding serum calcium levels, the mean level was higher in stable cases (8.6 ± 0.7) compared to unstable cases (8.4 ± 0.9), but the difference was not statistically significant (P-value = 0.540). Calcium levels were also similar between males (8.4 ± 0.8) and females (8.3 ± 0.9) (P-value = 0.417). The association between age and serum calcium level was weak and inversely related but not statistically significant (Pearson correlation coefficient: -0.038, P-value = 0.722) (Fig 1).

### Power analysis

The post-hoc power analysis revealed a power of 99.8% for the results of vitamin D, which exhibited a significant difference between study groups, and 55.7% for the results of calcium, showing non-significant differences between study groups. However, it is important to acknowledge that post-hoc power analysis has limitations and should be interpreted cautiously. While it offers insights into the adequacy of sample size and the likelihood of detecting significant effects, it is not considered the best practice for assessing the strength of a study.

## Discussion

This study investigated the levels of serum vitamin D and calcium and their association with the severity of IT fractures. The findings indicated significantly higher levels of vitamin D in patients with stable IT fractures compared to unstable cases. Although calcium levels were also higher in stable cases with IT fractures, the difference did not reach statistical significance. Additionally, age and sex were not notable predictors of the type of IT fractures regarding the assessed biomarkers in this study.

The findings presented in this study were comparable to similar literature. In a retrospective study on IT fractures in patients aged ≥60 years, including 21 cases of stable fracture and 36 cases of unstable fracture, with an overall mean age of 81.93 ± 9.58 years and females comprising 71.9% of the patients, the level of 25 (OH) vitamin D was significantly higher in the stable group (27.01 ± 15.75) compared to the unstable group (17.52 ± 8.65) (P-value = 0.010), which was similar to our study. However, in that investigation, the calcium level was also remarkably higher in the stable group (8.48 ± 0.45) rather than the unstable cases (8.19 ± 0.43) (P-value = 0.019), which was different from our findings [15].

Another cross-sectional study that included 114 patients with IT fractures, with 47.7% of them being male and an average age of 66 ± 16.67 in males and 71 ± 14.29 in females, categorized the IT fractures based on the Boyd & Griffin classification into four types. The results indicated a strong statistical association between the degree of vitamin D deficiency and the type of IT fracture based on the assumed classification [16]. In the mentioned study, different levels of vitamin D deficiency were observed in type Ⅱ fractures followed by type Ⅲ fractures; for example, severe vitamin D deficiency was only observed in these two types, with 66.7% of them having type Ⅱ fractures and 33.3% having type Ⅲ fractures [16].

In a series of 196 hip fracture cases, including 101 with femoral neck fractures and 95 cases with femoral IT fractures, female patients constituted the dominant proportion in both groups, comprising 80.20% of the first group and 57.89% of the second group. The mean age of patients with femoral neck fractures was 73.85 ± 11.88, and for the IT group, it was 76.27 ± 11.93. Serum calcium levels were assessed at the time of admission, post-operation, and discharge. The results indicated significantly higher values among patients with femoral neck fractures compared to IT cases in the admission (2.19 ± 0.12 vs. 2.13 ± 0.15, P-value = 0.002) and discharge phases (2.15 ± 0.13 vs. 2.10 ± 0.16, P-value = 0.019), but not in the post-operation assessment (2.01 ± 0.12 vs. 2.01 ± 0.14, P-value = 1.000) [17]. The authors of this study suggested that serum calcium preservation and restoration in patients with femoral neck fractures might be better than those with IT fractures based on their findings, and they proposed that low serum calcium might predispose patients to femoral IT fractures [17].

Other studies related to the theme of our investigation were found to compare the findings with a wider scope of patients, mostly focused on overall patients with hip fractures [13, 14, 19–22]. A recent prospective study on an elderly population aged > 50 years, which included 120 patients with a male dominance of 56.7%, found the most common type of fracture to be IT fracture (59.2%), followed by femoral neck fracture (29.2%) and subtrochanteric fracture (11.7%). The study assessed levels of serum calcium and vitamin D based on different types of hip fractures and their severity. The authors concluded that the more deficient the calcium and the more deficient/insufficient the vitamin D level, the more severe type of fracture was observed in each location [13]. The study found the mean level of serum calcium to be 8.83 mg/dl and vitamin D to be 21.85 ng/dl among patients with IT fractures, with a decreasing pattern of both biomarkers observed from type Ⅰ to Ⅳ of the Boyd & Griffin classification, with overall significant differences (P-value = 0.001) [13].

A cross-sectional study of 324 patients aged > 65 years, with a mean age of 83 ± 7 years and a female predominance (80%), found that vitamin D deficiency was more frequent and severe in patients with severe femoral neck fractures (Garden III–IV types) and IT fractures (Kyle III–IV) compared to those with less severe fractures (frequency: 74% vs. 57%, 25 (OH) vitamin D3 level: 20 ± 15 vs. 26 ± 21) [14]. The investigators of that study suggested vitamin D supplementation in the elder population to prevent severe femoral neck and IT fractures, as the reported findings were indicative of an association between vitamin D deficiency and the severity of osteoporotic hip fractures [14].

A study on the prevalence of vitamin D deficiency in Asian-Indian patients with fragility hip fractures reported a high prevalence, with 96.7% having vitamin D deficiency, and found significantly lower bone mass density (BMD) in these patients compared to age- and sex-matched healthy controls [19]. Another investigation on 1319 subjects aged 65–88 years old revealed overall higher BMD values in groups with higher serum 25 (OH) vitamin D levels and suggested a cut-off of 50–60 nmol/liter for this element, where values higher than this range might be associated with improved bone health and physical activity [20]. In a prospective study on 360 patients with fragility hip fractures, with a mean age of 84.7 ± 8.2 years and mostly being female (77.8%), vitamin D was insufficient (<30 ng/mL) in 93.9% and deficient (<20 ng/mL) in 7.17% of the patients, with the values of serum 25 (OH) vitamin D being higher in the younger population [21].

Vitamin D deficiency might be associated with lower BMD, which is important in the prevention of fragility fractures of the hip [23]. However, it is proven in the literature that vitamin D supplementation in the prevention of hip fractures risk is only effective if calcium supplementation is prescribed for patients [10, 11, 24]. Additionally, low vitamin D serum levels have been associated with an increased risk of non-orthopedic complications in patients with hip fractures, including the risk of delirium, refractures of the hip, and readmissions due to other medical complaints [12]. Compiling the findings of the referred studies could suggest vitamin D supplementation as a preventive measure against fragility fractures and more severe types of fractures, specifically in IT fractures as a common type of hip fracture with a high burden in the elder population (Table 2).

**Table 2:** Summary of previous studies in vitamin D and intertrochanteric fractures association and results of our study.

Study	Population Characteristics	Key Findings on Vitamin D Levels	Key Findings on Calcium Levels	Other Key Findings
Hwang et al.	Retrospective study, 57 patients aged ≥60 years, mean age 81.93 ± 9.58 years, 71.9% female	Stable IT fractures had higher vitamin D (27.01 ± 15.75) compared to unstable fractures (17.52 ± 8.65) (P = 0.010)	Stable fractures had higher calcium (8.48 ± 0.45) than unstable fractures (8.19 ± 0.43) (P = 0.019)	N/A
Jamal et al.	Cross-sectional study, 114 patients with IT fractures, 47.7% male, average age 66 ± 16.67 (males), 71 ± 14.29 (females)	Severe vitamin D deficiency observed in type II (66.7%) and type III (33.3%) IT fractures	N/A	Used Boyd & Griffin classification for IT fractures
Li et al.	Series of 196 hip fracture cases (101 femoral neck, 95 IT fracture), mean age 73.85 ± 11.88 (femoral neck), 76.27 ± 11.93 (IT), 80.20% female (femoral neck), 57.89% female (IT)	N/A	Higher calcium in femoral neck rather than IT fractures at admission (2.19 ± 0.12 vs. 2.13 ± 0.15, P = 0.002) and discharge (2.15 ± 0.13 vs. 2.10 ± 0.16, P = 0.019), but not post-operation (2.01 ± 0.12 vs. 2.01 ± 0.14, P = 1.000)	Suggested serum calcium preservation is better in femoral neck fractures
Hublikar et al.	Prospective study, 120 patients >50 years, 56.7% male. 59.2% IT fracture, 29.2% femoral neck fracture and 11.7% subtrochanteric fracture	Vitamin D level 21.85 ng/dl in IT fractures	Mean calcium level 8.83 mg/dl in IT fractures	significant decrease of both biomarkers from type I to IV Boyd & Griffin classification (P = 0.001)
Larrosa et al.	Cross-sectional study, 324 patients >65 years, mean age 83 ± 7 years, 80% female	Vitamin D deficiency more frequent in severe femoral neck (Garden III-IV) and IT (Kyle III-IV) fractures (74% vs. 57%, 25 (OH) vitamin D3: 20 ± 15 vs. 26 ± 21)	N/A	Suggested vitamin D supplementation in the elder population to prevent severe fractures
Niikura et al.	Prospective study, 360 patients with fragility hip fractures, mean age 84.7 ± 8.2 years, 77.8% female	93.9% had vitamin D insufficiency (<30 ng/mL), 7.17% had vitamin D deficiency (<20 ng/mL)	N/A	N/A
Our Study	286 patients with IT fractures, mean age 70.6 years, 48.6% female	Stable fractures: 30.3 ± 7.0 ng/ml; Unstable fractures: 26.5 ± 6.1 ng/ml (P = 0.007)	Stable fractures: 8.6 ± 0.7 mg/dl; Unstable fractures: 8.4 ± 0.9 mg/dl (P = 0.540)	Stable fractures had significantly higher vitamin D levels compared to unstable fractures. No significant difference in calcium levels.

IT: Intertrochanteric fracture

In addition to reducing the incidence of intertrochanteric fractures as reported in the literature, our findings indicate that adequate vitamin D levels can mitigate fracture severity and the risk of unstable fractures. Therefore, screening and maintaining normal vitamin D levels through supplementation is crucial.

The current study had some limitations. Lack of multiple assessments of serum calcium and vitamin D levels, lack of evaluation of other related biomarkers like parathyroid hormone, lack of data on patients’ comorbidities and drug histories. Additionally, the study’s limitations were compounded by the non-random selection of patients and its conduct in only two centers, which could have introduced laboratory variations and biases into the results. On the other hand, this study had major strengths and achievements as it was among the first efforts to study this notion in Iran and via a multicenter design. Future similar studies are suggested to validate the findings of this study and provide evidence for future possible trials to prove the efficacy and safety of calcium and vitamin D supplementation for the prevention of severe hip fractures, including IT fractures. Regarding the potential of serum vitamin D and calcium supplementation for IT fracture prevention, further research is warranted to explore this aspect comprehensively. However, our study lays a foundation for future investigations in this area.

## Conclusions

Findings of this study showing higher levels of serum calcium and vitamin D in patients with stable IT fractures compared to unstable cases suggest these two elements as possible effective and protective agents against more severe IT fractures, as a common type of hip fractures.
